# Supplementary material for: Lipopolyplex-Mediated Co-Delivery of Doxorubicin and FAK siRNA to Enhance Therapeutic Efficiency of Treating Colorectal Cancer
Source: Pharmaceutics. 2023 Feb 10;15(2):596. doi: 10.3390/pharmaceutics15020596 (PMC9968081; doi:10.3390/pharmaceutics15020596)
Supplement: Supplementary file 1 [file pharmaceutics-15-00596-s001.zip › pharmaceutics-2134079-supplementary.pdf]

pharmaceutics

Supplementary Materials: Lipopolyplex-Mediated Co-Delivery of Doxorubicin and FAK siRNA to Enhance Therapeutic Efficiency of Treating Colorectal Cancer

Tilahun Ayane Debele, Chi-Kang Chen, Lu-Yi Yu and Chun-Liang Lo

Synthesis and identification of MABH monomer

The synthesis of monomeric MABH was achieved using the methacrylic chloride (MACl) with tert-butyl carbazate (Boc-hydrazide). Briefly, the Boc-hydrazide and 1.5 times the molar Triethylamine (TEA) placed in a two-necked flask, dissolved under a nitrogen atmosphere, and uniformly mixed with DCM. After dissolving MACl in DCM, it was added drop wise into a two-necked flask through a feeding tube and reacted for 12 hours. After completion of the reaction, most of the TEA·HCl salts were first removed by gravity filtration followed by sequential extraction with 0.1 N HCl (aq), saturated $NaHCO_3$ (aq) and saturated brine to remove excess MACl and TEA. The resulting organic solution layer was removed with $MgSO_4$, the solids were removed by filtration, the organic solution was removed by rotary evaporation and finally a white solid was obtained after recrystallization from a solution of Hexane (v: v = 1: 1) in ethyl acetate. The dried product was identified by ^{1}H-NMR and FT-IR. For ^{1}H-NMR, 5-6 mg / ml of the sample was dissolved with DMSO-d_6 and analyzed by proton nuclear magnetic resonance spectroscopy. The FT-IR sample is obtained by dissolving the product in DCM and analyzing the chemical structure by the thin film method.

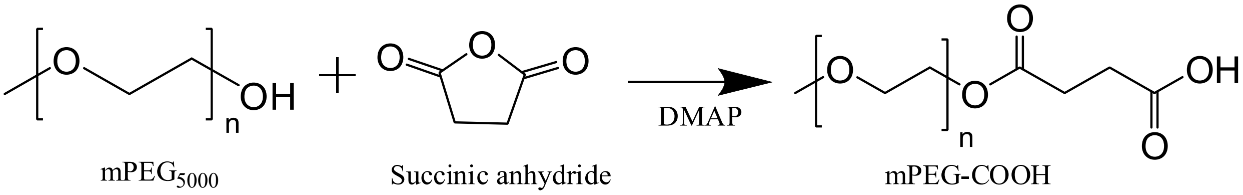

Scheme S1. mPEG5000-COOH modification steps.

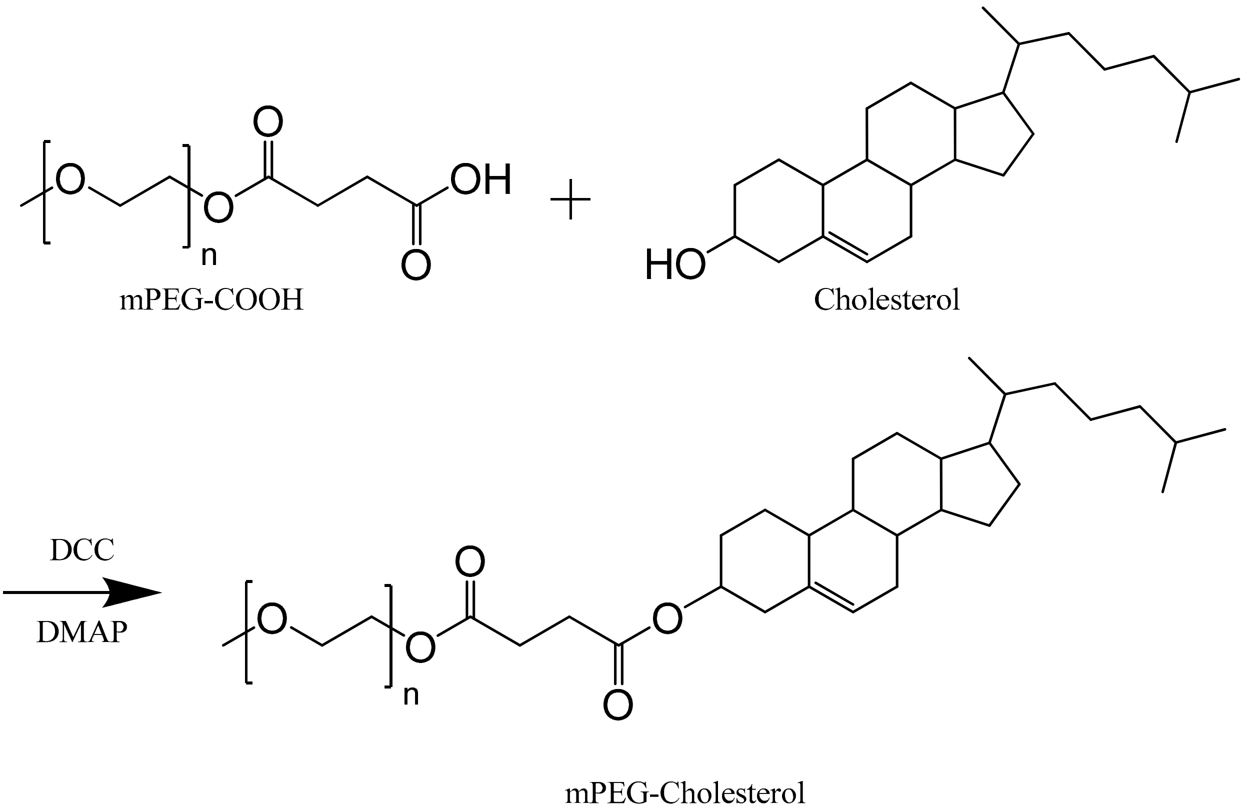

Scheme S2. Synthesis of mPEG5000-cholesterol.

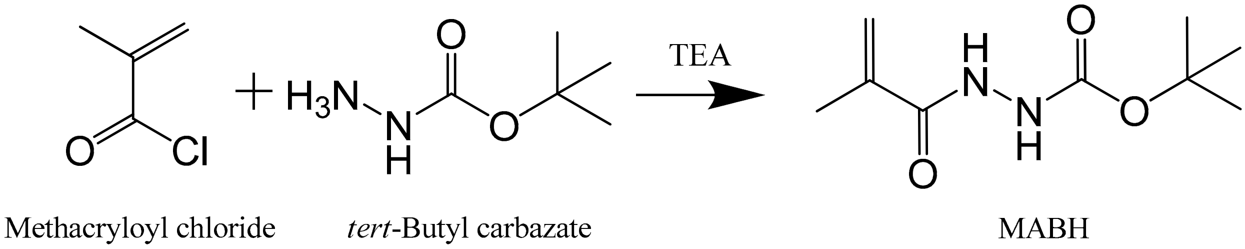

Scheme S3. Synthesis of free radical monomer MABH.

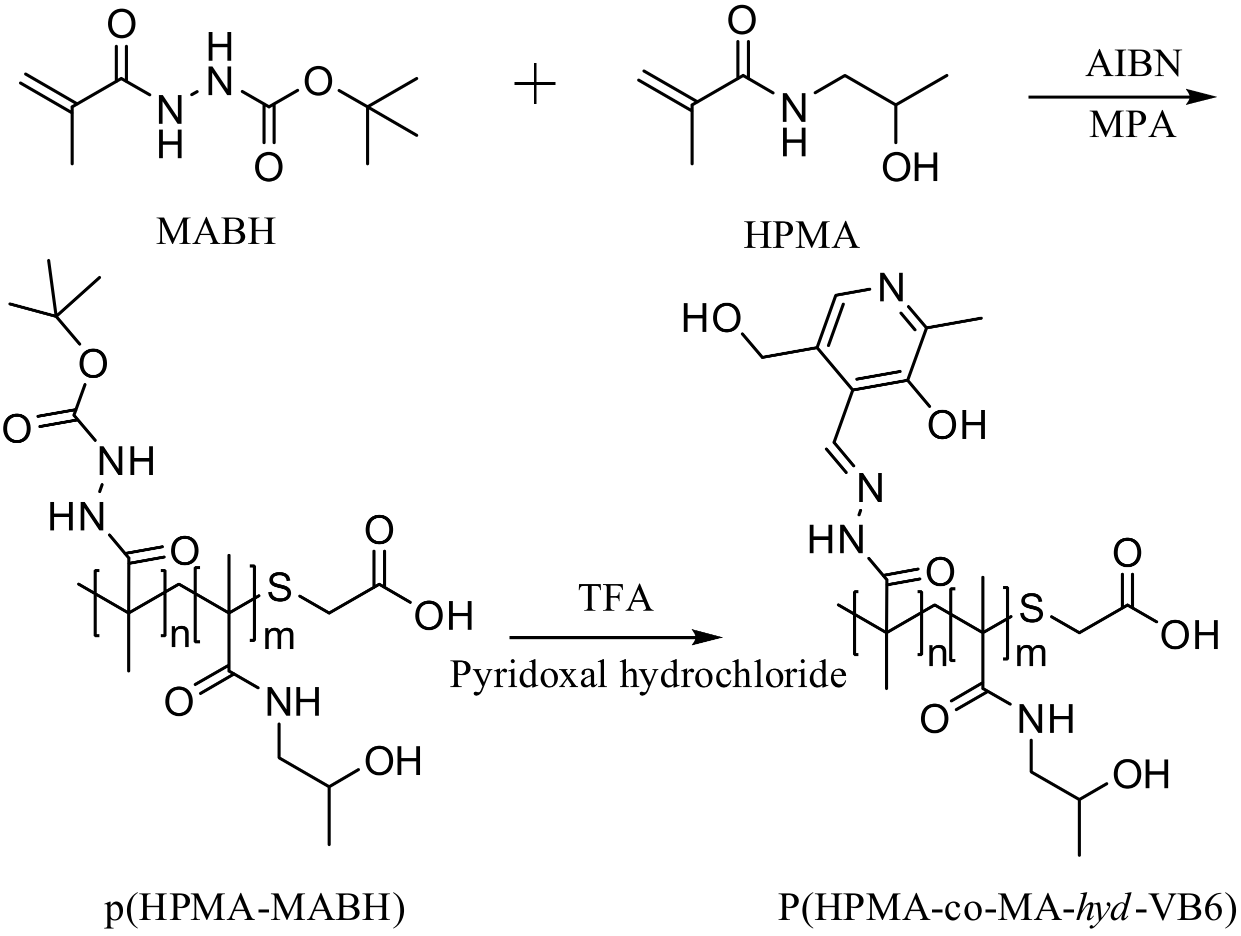

Scheme S4. Synthesis of P(HPMA-co-MA-*hyd*-VB6) copolymers.

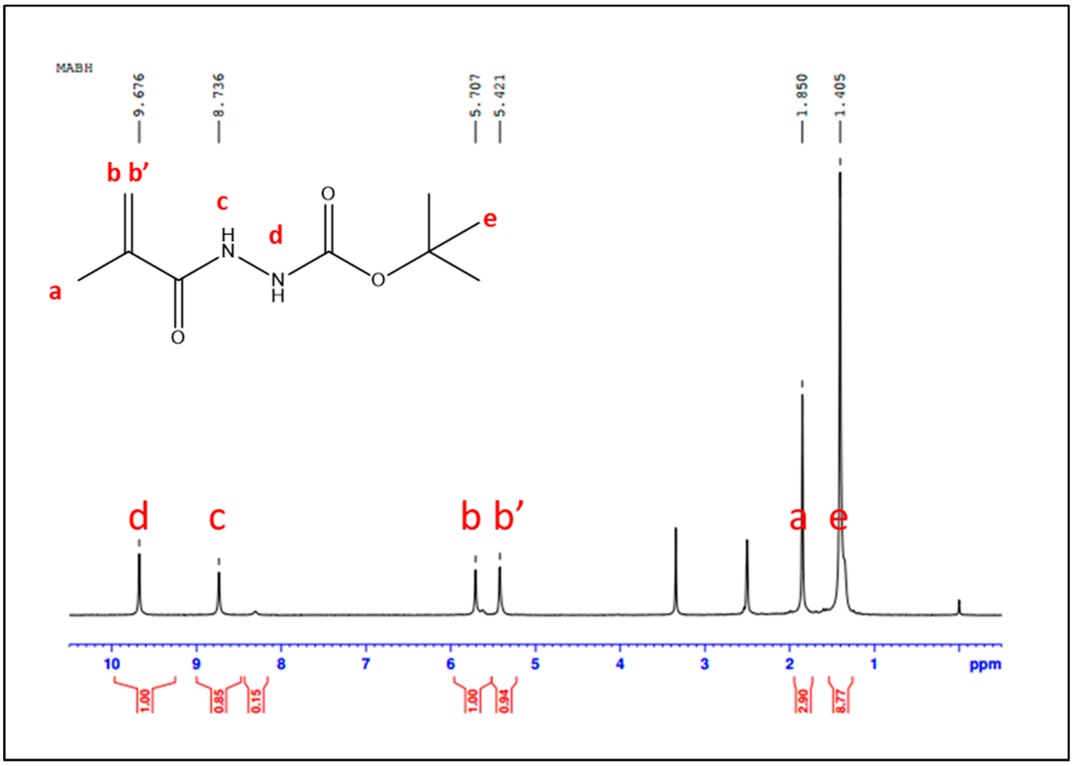

Figure S1. ^{1}H-NMR spectra of monomer MABH.

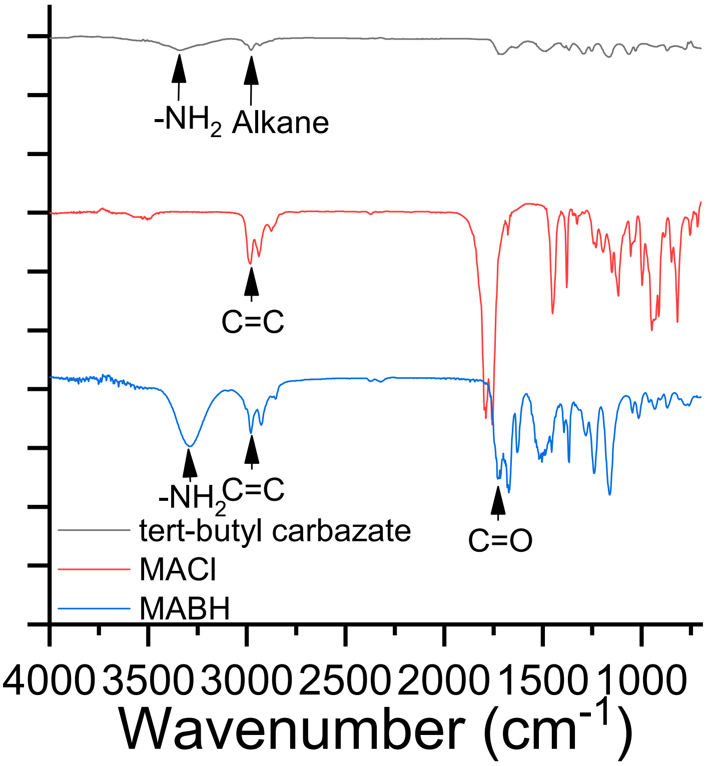

Figure S2. FT-IR spectrum of monomer MABH.

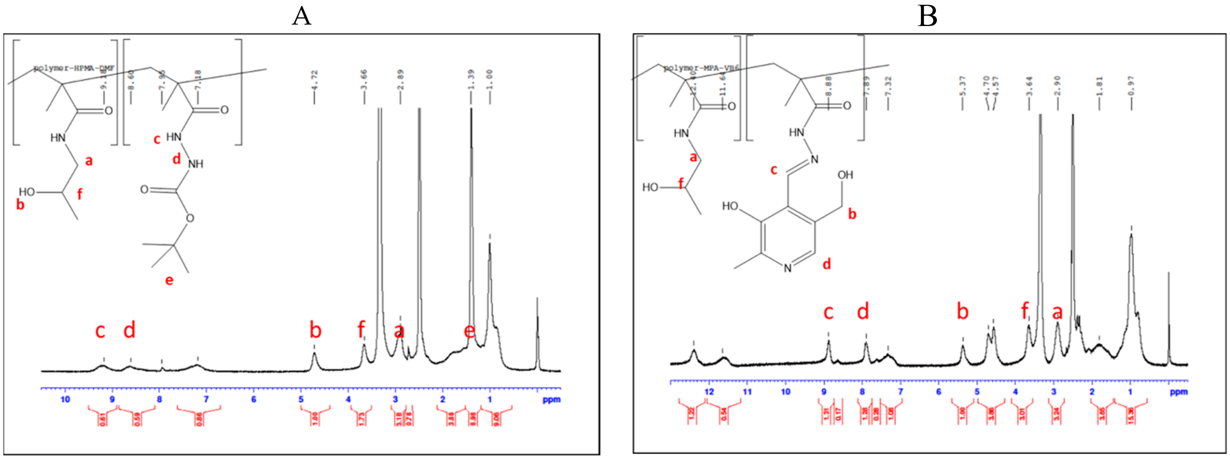

Figure S3. ^{1}H-NMR spectrum of (A) P(HPMA-*co*-MABH) copolymers and (B) P (HPMA-*co*-MA-*hyd*-VB6) copolymers.

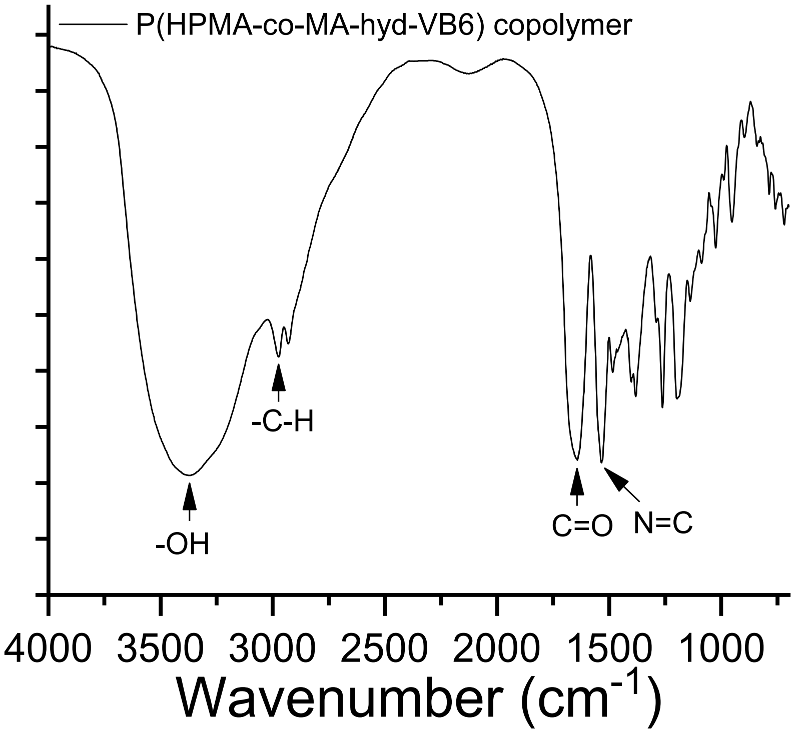

Figure S4. FT-IR spectrum of P(HPMA-*co*-MA-*hyd*-VB6) copolymers.

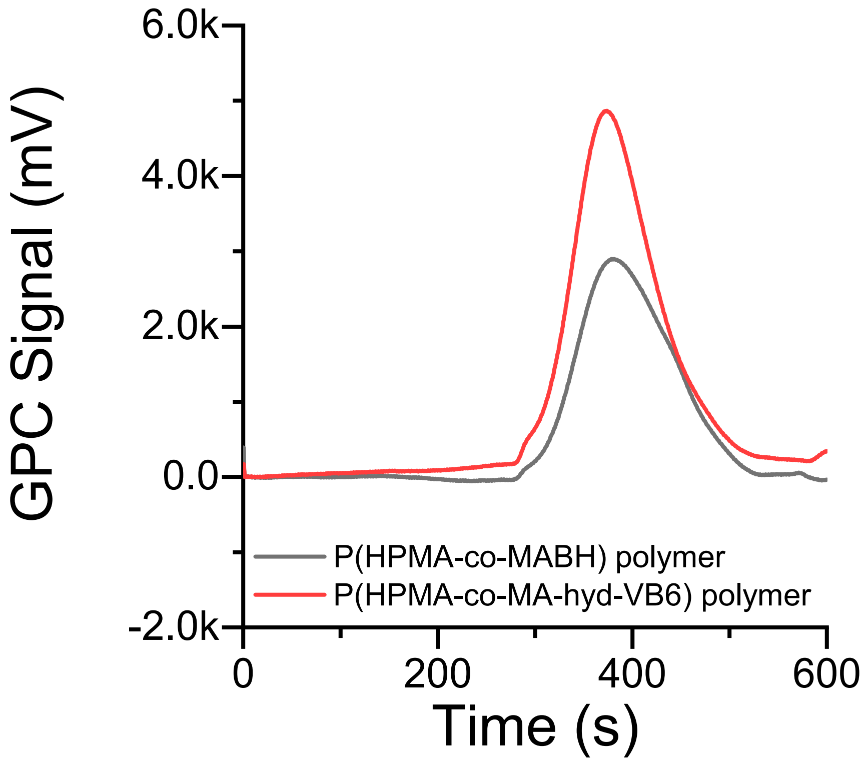

Figure S5. The GPC chromatography of P(HPMA-*co*-MABH) and P(HPMA-*co*-MA-*hyd*-VB6) copolymers.

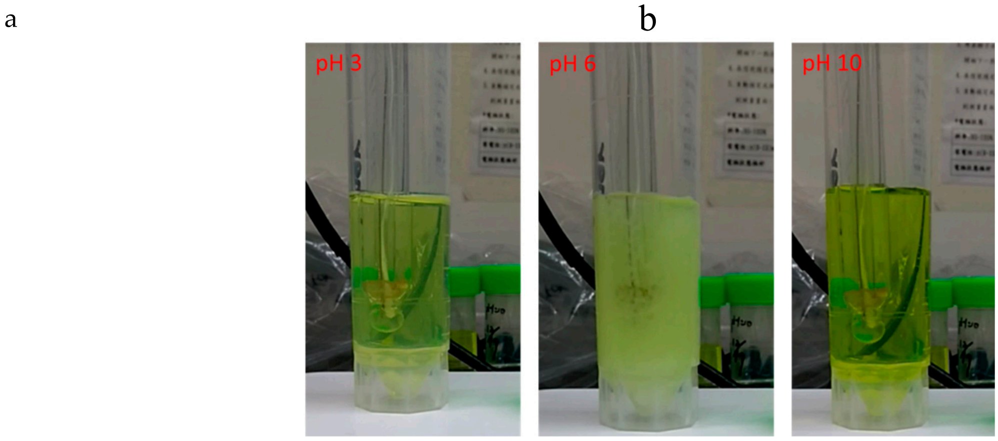

Figure S6. The proton absorption test of P10K45 copolymers. (a) The titration of P10K45 copolymers treated with 0.05 N NaOH. (b) The phase changes of copolymer solution at pH 3, pH 6 and pH 10.

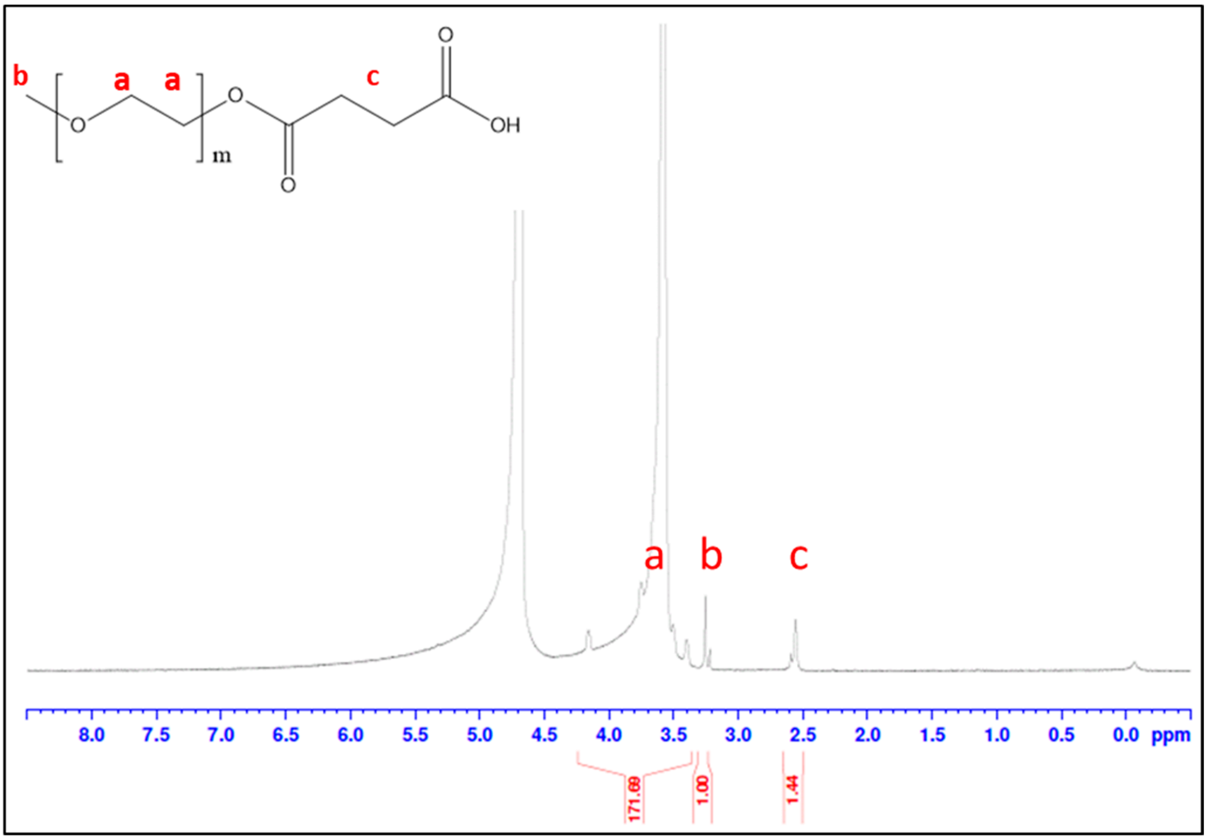

Figure S7. ^{1}H-NMR spectrum of mPEG-COOH. ^{1}H NMR mPEG-COOH (D_2O): δ 2.5-2.6 (-C<u>H</u>$_2$-C<u>H</u>$_2$- from succinic acid); δ 3.2-3.3 (-O-C<u>H</u>$_3$ from mPEG5000); δ 3.4-4.2 (-O-C<u>H</u>$_2$-C<u>H</u>$_2$-O- from mPEG5000).

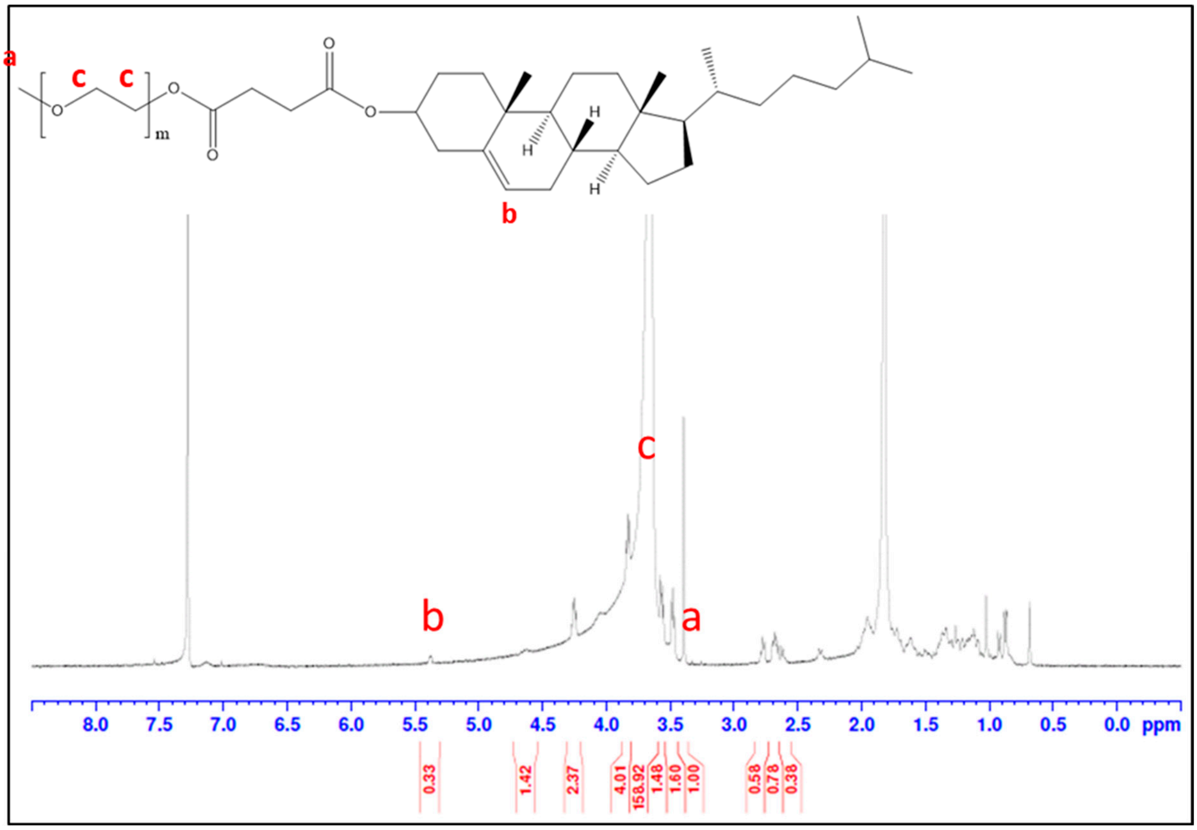

Figure S8. ^{1}H-NMR spectrum of mPEG-cholesterol. ^{1}H-NMR ($CDCl_3$) of mPEG-cholesterol δ-3.2-3.3 (-O-CH3 from mPEG5000); δ 3.4-4.2 (-O-CH2-CH2-0- from mPEG5000); δ 5.3-5.5 (-CH = CH2- from cholesterol).

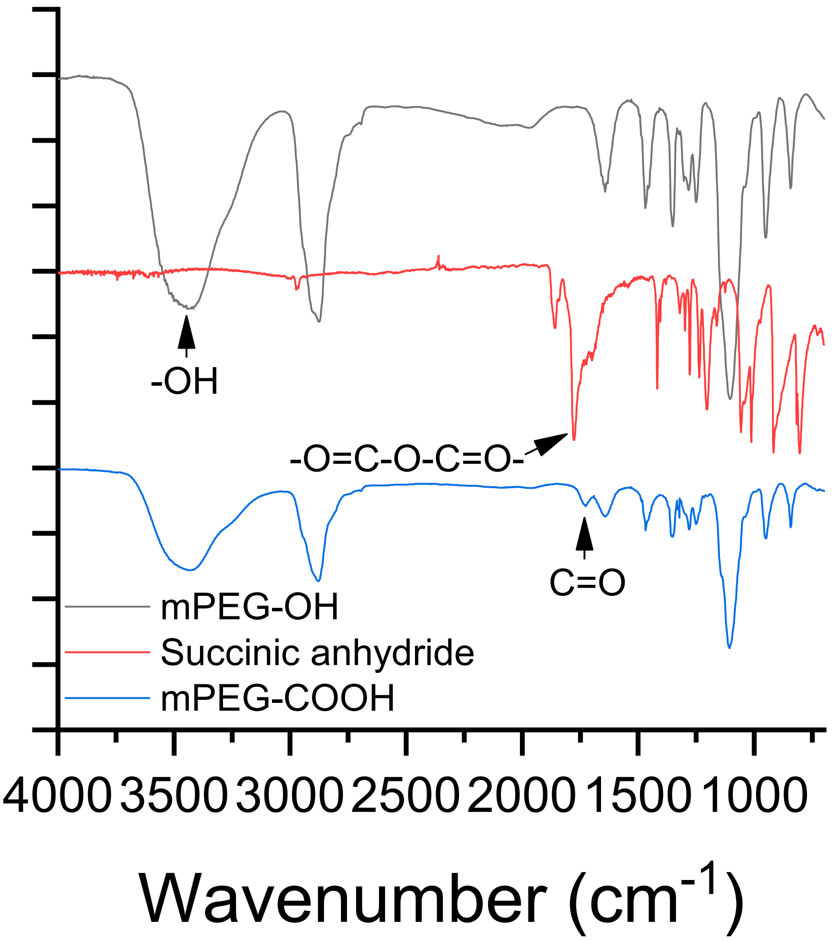

Figure S9. FT-IR spectrum of mPEG-COOH. FT-IR of mPEG-COOH at 1700 cm^{-1} (-C-CO-O-CO-C-bond); 3500 cm^{-1} (-OH bond).

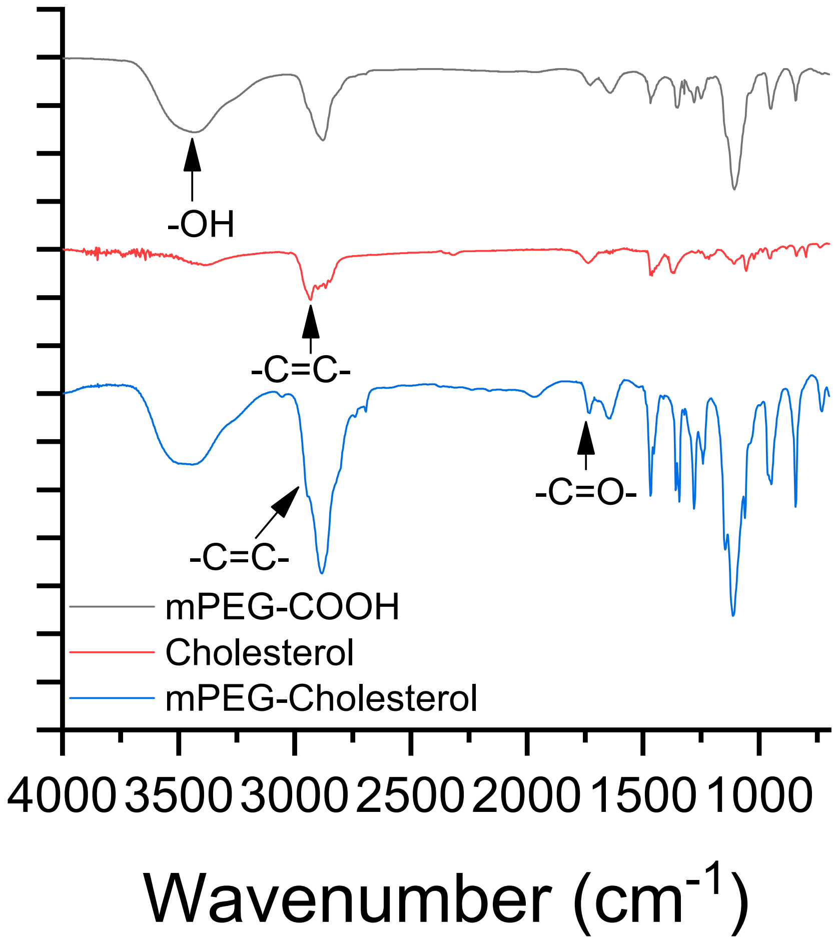

Figure S10. FT-IR spectra of mPEG-cholesterol. mPEG-cholesterol FT-IR at 3000 cm^{-1} (C = C bond); 3400 cm^{-1} (-OH bond).

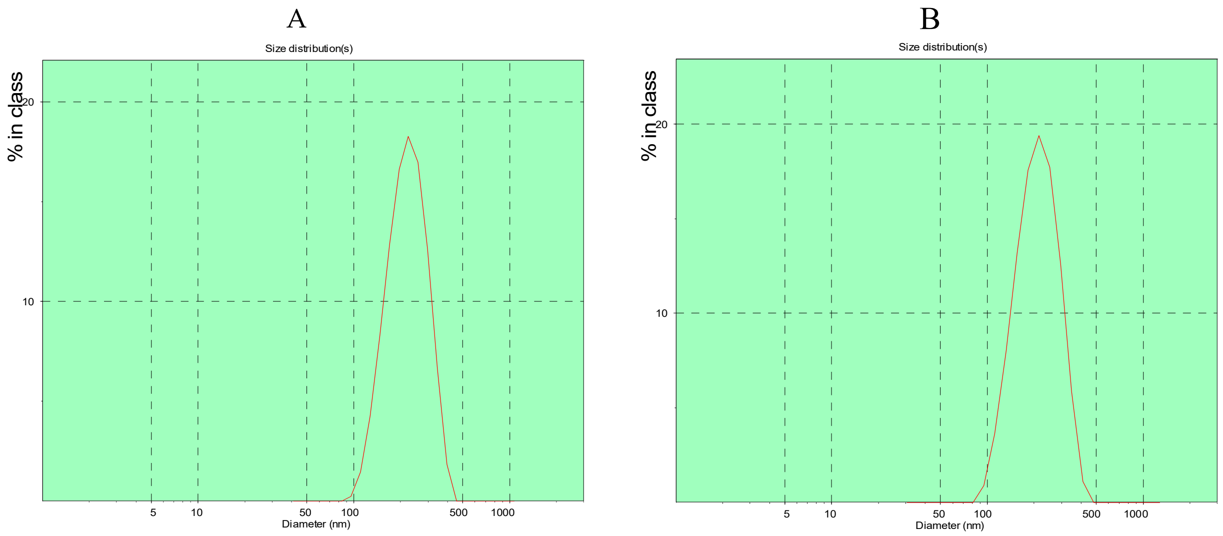

Figure S11. The particle size distribution of (A) LP and (B) DLP lipopolyexes.

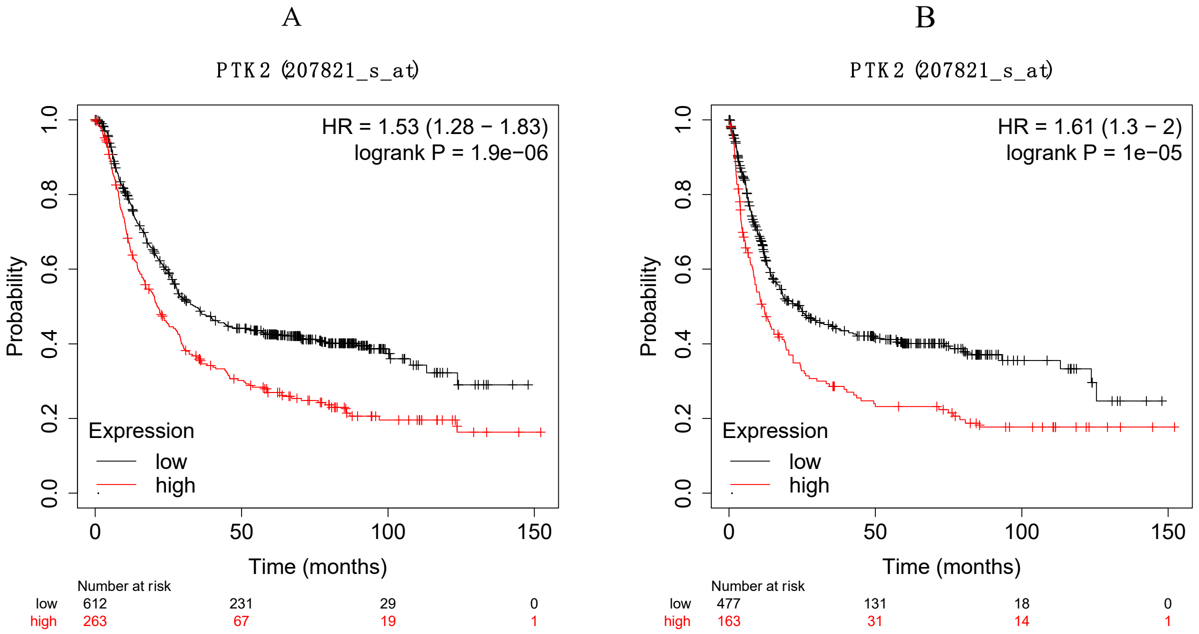

Figure S12. The Kaplan–Meier plot of progression-free survival (PFS) and overall survival (OS) according to FAK expression status in gastric cancer patients. The p-value for the differences between the two curves was determined using the log-rank test. (A) OS for the overall population. (n = 875, log-rank test: p = 1.9e−06) (B) PFS for the overall population. (n = 640, log-rank test: p = 1e−05).
